# Neprilysin inhibition in chronic kidney disease

**DOI:** 10.1093/ndt/gfu269

**Published:** 2014-08-18

**Authors:** Parminder Judge, Richard Haynes, Martin J. Landray, Colin Baigent

**Affiliations:** Clinical Trial Service Unit and Epidemiological Studies Unit (CTSU), Nuffield Department of Population Health, University of Oxford, Oxford OX3 7LF, UK

**Keywords:** cardiovascular disease, chronic kidney disease, heart failure, hypertension, neprilysin inhibition

## Abstract

Despite current practice, patients with chronic kidney disease (CKD) are at increased risk of progression to end-stage renal disease and cardiovascular events. Neprilysin inhibition (NEPi) is a new therapeutic strategy with potential to improve outcomes for patients with CKD. NEPi enhances the activity of natriuretic peptide systems leading to natriuresis, diuresis and inhibition of the renin–angiotensin system (RAS), which could act as a potentially beneficial counter-regulatory system in states of RAS activation such as chronic heart failure (HF) and CKD. Early NEPi drugs were combined with angiotensin-converting enzyme inhibitors but were associated with unacceptable rates of angioedema and, therefore, withdrawn. However, one such agent (omapatrilat) showed promise of NEP/RAS inhibition in treating CKD in animal models, producing greater reductions in proteinuria, glomerulosclerosis and tubulointerstitial fibrosis compared with isolated RAS inhibition. A new class of drug called angiotensin receptor neprilysin inhibitor (ARNi) has been developed. One such drug, LCZ696, has shown substantial benefits in trials in hypertension and HF. In CKD, HF is common due to a range of mechanisms including hypertension and structural heart disease (including left ventricular hypertrophy), suggesting that ARNi could benefit patients with CKD by both retarding the progression of CKD (hence delaying the need for renal replacement therapy) and reducing the risk of cardiovascular disease. LCZ696 is now being studied in a CKD population.

## INTRODUCTION

Patients with chronic kidney disease (CKD) face many hazards including increased risk of progression to end-stage renal disease (ESRD) and premature mortality from cardiovascular disease (CVD) [1, 2]. Whereas a minority of patients with CKD will reach ESRD, CVD is much more common. A variety of processes contribute to this excess risk including atherosclerosis, arteriosclerosis, hypertension, sympathetic hyperactivity and structural heart disease [including left ventricular (LV) hypertrophy], which may manifest clinically as heart failure (HF) [2]. As CKD progresses, the contribution of atherosclerosis becomes proportionally smaller and arteriosclerosis and structural heart disease predominate, potentially explaining the high incidence of sudden cardiac death in patients with advanced CKD [2]. The similarities in the manifestation of CVD observed in patients with advanced CKD and that in patients with HF raises the hypothesis that treatments proven to be effective in the HF population may also be beneficial in patients with advanced CKD. However, such patients have not been studied in randomized cardiological trials.

Randomized trials have shown that renin–angiotensin system (RAS) inhibitors [RASi; angiotensin-converting enzyme inhibitors (ACEi) and angiotensin receptor blockers (ARB)] reduce the risk of ESRD in patients with diabetic and non-diabetic proteinuric CKD [3–6]. In the general population, RASi reduce cardiovascular events, and meta-analyses suggest that the mechanism of this benefit is not simply blood pressure (BP) reduction [7, 8]. However, trials of RASi in patients with advanced CKD have not shown benefits on cardiovascular outcomes, although this may be because they were not large enough to do so [9].

Although dual ACEi/ARB therapy reduces albuminuria more than either agent alone, trials have shown that this does not translate into either cardiovascular benefit or additional renal protection [10–13]. Indeed, in those trials, dual therapy was associated with increased risk of adverse effects including hyperkalaemia and acute kidney injury [11–13]. Similar outcomes were observed when RASi was combined with a direct renin inhibitor (aliskiren) as an alternative approach to dual RASi [14].

The lack of benefit associated with dual RAS blockade highlights the need for new therapeutic strategies in CKD. The natriuretic peptide (NP) system is a neurohormonal system that counter-regulates the RAS. Therefore, enhancing the activity of NPs may be beneficial in states of RAS activation, such as cardiovascular and kidney disease.

## NP SYSTEM AND NEPRILYSIN

**FIGURE 1:**
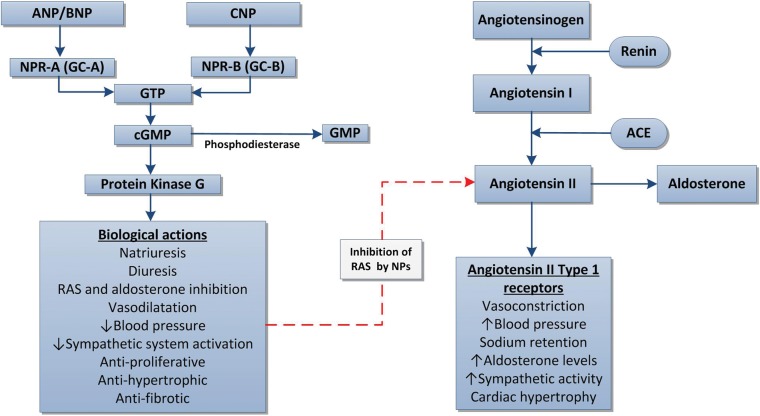
Mechanism of action of NPs [16, 17]. GTP, Guanosine-5′-triphosphate.

NPs are a family of three peptides that include atrial, brain and c-type NPs (ANP, BNP and CNP, respectively) [15]. ANP and BNP are predominantly synthesized and released from cardiac myocytes in response to atrial stretch due to raised venous pressure. ANP precursor expression in the kidney produces a subtype called urodilatin from distal tubular cells, whereas CNP is predominantly expressed in endothelial cells [15, 16]. All three NPs are formed as pre-pro-peptides and undergo several cleavage steps to form active peptides. NPs exert physiological effects via NP receptors (NPRs). ANP and BNP act via NPR-A (guanylate cyclase-A) and CNP via NPR-B (guanylate cyclase-B) [17]. These receptors are coupled to cyclic guanosine monophosphate (cGMP)-dependent signalling (Figure 1) [15–17].

ANP and BNP have a range of renal and cardiovascular effects contributing to natriuresis, diuresis and BP regulation [16, 17]. CNP is a vasoactive peptide with marked cardiovascular effects but minimal renal actions [16, 17]. Both ANP and urodilatin regulate renal sodium and water excretion by inhibition of angiotensin II- and aldosterone-dependent sodium and water reabsorption and inhibition of antidiuretic hormone [17]. Natriuresis results from afferent arteriolar vasodilatation and efferent arteriolar vasoconstriction, increasing renal vascular resistance and glomerular filtration. ANP also causes relaxation of mesangial cells, further increasing the capillary surface area for filtration and hence diuresis [18]. In addition, ANP inhibits endothelin production, proliferation of smooth muscle cells and myocardial hypertrophy [17, 18].

Animal models lacking the proANP gene develop salt-sensitive hypertension [19]. Gene delivery of ANP to mice with salt-sensitive hypertension reduces BP, cardiac hypertrophy, stroke and renal injury [20, 21]. Recently, two single nucleotide polymorphisms rs5068 and rs1938358 in the ANP and BNP genes have been found to be associated with both increased levels of NT-proANP and NT-proBNP, respectively, and with lower BP and an improved metabolic profile [22]. These genetic data suggest that augmenting NP concentrations could lead to improved clinical outcomes.

### Neprilysin

Neprilysin [also known as neutral endopeptidase (NEP)] is the key enzyme responsible for degradation of NPs [17]. NEP is a membrane-bound zinc-containing metalloproteinase with widespread tissue distribution including the brain, vascular endothelial cells, smooth muscle cells, cardiac myocytes and neutrophils, but has greatest abundance in the brush border of proximal renal tubular cells [16, 23]. NEP is also responsible for processing and catabolism of a range of other vasoactive peptides including bradykinin, substance P, angiotensin II and endothelin [23].

The broad range of potential therapeutic actions of NPs led to development of agents that inhibit NEP. Neprilysin inhibition (NEPi) results in potent natriuresis and vasodilation; in the kidney, this vasodilatory effect reduces intraglomerular pressure and proteinuria [24, 25]. Chronic isolated NEPi does not translate into clinically meaningful BP reductions as NEPi impairs breakdown of angiotensin II and any BP effects are offset by up-regulation of RAS and sympathetic nervous system activity. The beneficial renal and cardiovascular effects of NEPi are enhanced when combined with RASi and this has led to development of dual NEPi/RASi [16].

## DUAL NEPi/ACEi (VASOPEPTIDASE INHIBITORS)

Dual NEPi/ACEi are also known as vasopeptidase inhibitors (VPIs). Many compounds have been produced and trialled in humans (Table 1). Omapatrilat was the most studied VPI.

**Table 1.** VPIs produced and studied in humans

VPI	Situation studied	Year
MDL-100240	Healthy volunteers	2000
Sampatrilat	Hypertension	1998–99
Fasidotril	Hypertension	2000
Omapatrilat (BMS-186716)	Hypertension, HF and CVD	1999–2004

Omapatrilat was well tolerated in studies of healthy volunteers among whom it significantly increased urinary excretion of ANP and cGMP (i.e. markers of NEPi). Omapatrilat also produced potent ACE inhibition with decreased levels of angiotensin II and reduced systemic BP. Renal effects of omapatrilat included marked increases in renal blood flow without associated change in glomerular filtration rate (GFR) and decreased filtration fraction. This haemodynamic profile could translate into renal protection and slower progression of CKD, as discussed further below [24, 25].

### VPIs and angioedema

Despite the promising cardiorenal and neurohormonal findings seen with VPIs, omapatrilat was associated with excess rates of angioedema. In 723 patients with CVD, omapatrilat reduced BP but six cases of angioedema occurred [26]. In the Omapatrilat Cardiovascular Treatment versus Enalapril (OCTAVE) trial, angioedema occurred with greater severity and frequency with omapatrilat than enalapril [274/12 609 (2.17%) versus 86/12 557 (0.68%); relative risk 3.17; 95% confidence interval (95% CI) 2.52–4.12; P < 0.005] [27]. Two of the participants experienced airway compromise, one of whom required mechanical ventilation. The mechanism of angioedema was found to be related to increased bradykinin activity with combined NEPi and ACEi (described below).

**FIGURE 2:**
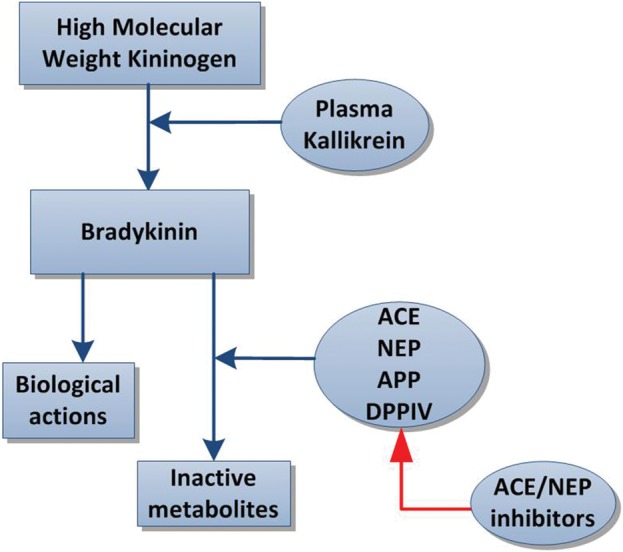
Mechanism of bradykinin action and inactivation by neprilysin.

Angioedema is an uncommon complication of ACEi therapy which is seen in 0.1–0.3% of treated patients and can occur at any interval after starting these drugs [28]. It can very rarely cause laryngeal oedema and asphyxiation leading to death [16]. ACE inhibitor-induced angioedema is thought to be mediated by decreased bradykinin breakdown resulting in increased bradykinin levels (Figure 2) [28, 29]. In an acute episode of angioedema, bradykinin concentrations can rise >10-fold [28].

Given the low incidence of angioedema associated with ACEi, it is thought that individuals are only affected if they have an additional risk factor, such as smoking (due to reduced NEP and dipeptidyl peptidase IV activity in smokers), black race (due to ACE gene polymorphisms) or hereditary angioedema (for example due to C1 inhibitor deficiency) [29, 30]. With ACE inhibition, bradykinin degradation becomes dependent on secondary enzymes (including NEP) for its breakdown and hence combined NEPi/ACEi had an additive effect on bradykinin levels. Following the results of the OCTAVE trial, the Food and Drug Administration review board did not approve omapatrilat and it was withdrawn from development by the manufacturer [16].

## DUAL NEP/ARB INHIBITION

**FIGURE 3:**
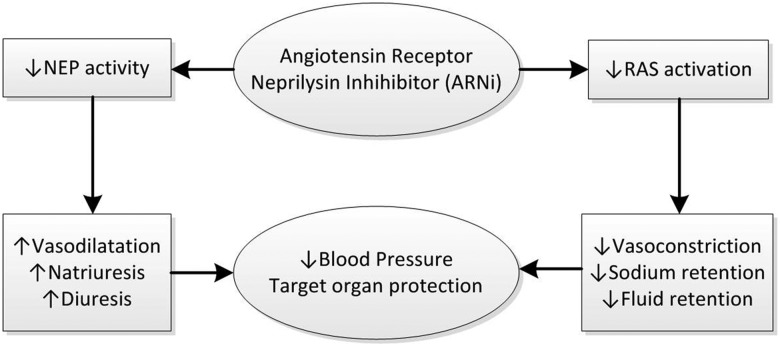
Mechanism of action of ARNi.

Whilst ACEi induce RAS blockade by inhibiting the conversion of angiotensin I to angiotensin II, angiotensin II receptor blockers (ARBs) elicit similar effects by blocking the activation of angiotensin II Type 1 receptors by angiotensin II. However, ARBs have minimal effect on bradykinin activity and, therefore, are much less likely to cause angioedema. This led to the development of dual-acting angiotensin receptor neprilysin inhibitors (ARNi), which combine the beneficial effects of ARBs and NEPi without excess risk of angioedema (Figure 3). LCZ696 was the first ARNi to be developed. It combines two drugs: an ARB moiety (valsartan) and an NEP inhibitor pro-drug (AHU377) in a 1:1 molar complex. Oral administration of LCZ696 delivers systemic exposure to the two separate moieties. AHU377 has a relatively short half-life and undergoes further rapid conversion by enzymatic cleavage of its ethyl ester to form the active NEPi compound, LBQ657 [31, 32].

In studies of healthy volunteers, AHU377 reached peak plasma concentrations in 0.5–1.1 h and the active moiety LBQ657 in 1.8–3.5 h [32]. LCZ696 was associated with increases in plasma cGMP, renin and angiotensin II levels. Systemic exposure to valsartan following dosing with LCZ696 demonstrated bioequivalence [e.g. 400 mg LCZ696 (maximum dose) is equivalent to 320 mg of valsartan] [32]. The drug was well tolerated in these participants [32, 33].

## NEPi IN HYPERTENSION

NEPi was originally studied using VPIs in a range of animal models of hypertension including salt-sensitive hypertension, stroke-prone spontaneous hypertensive rats and renovascular hypertension.

In the OCTAVE trial involving 25 302 hypertensive patients [27], compared with enalapril, at 8 weeks omapatrilat reduced systolic BP (SBP) by 3.6 mmHg (95% CI 2.6–4.6; P<0.001) and by 24 weeks fewer participants required adjunctive anti-hypertensive therapies (19 versus 27%, P < 0.001) [27].

**FIGURE 4:**
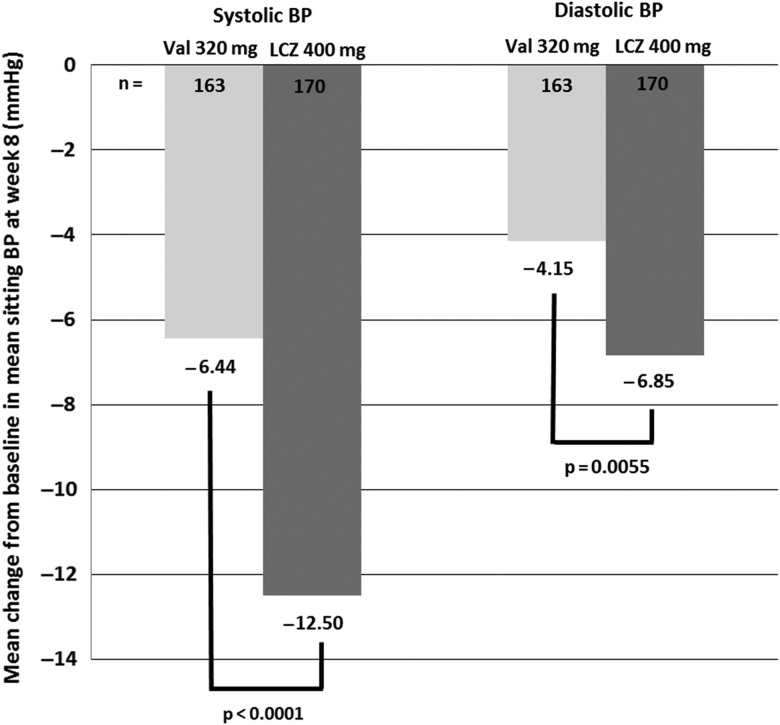
Difference in mean sitting SBP and DBP at week 8 (mmHg) [33].

A trial of 1328 hypertensive patients compared increasing doses of LCZ696 (100, 200 and 400 mg), valsartan (80, 160 and 320 mg), AHU377 (200 mg) or placebo [33]. The primary end point was mean change from baseline in mean sitting diastolic BP (DBP) between LCZ696 and valsartan during the 8-week treatment period. At the end of 8 weeks, the three LCZ696 doses had superior DBP lowering (mean reduction 2.17 mmHg; 95% CI 1.06–3.28; P < 0.0001) compared with the appropriate comparator dose of valsartan [33]. Single-dose pairwise comparisons showed that each dose of LCZ696 had greater SBP and DBP lowering than its equivalent dose of valsartan, and that the proportional reduction in SBP and of DBP increased with increasing LCZ696 dosage [results for mean change in SBP and DBP for LCZ696 (LCZ) 400 mg versus valsartan (Val) 320 mg are shown in Figure 4].

Plasma ANP and cGMP levels increased significantly with LCZ696. LCZ696 reduced albuminuria more than placebo, but not more than the equivalent dose of valsartan [33]. However, baseline albuminuria was low (geometric mean between 1.1 and 1.5 mg/mmol in all treatment groups). LCZ696 was well tolerated and no cases of angioedema occurred [33].

A recent trial has demonstrated similar efficacy in Asian patients (from Japan, China, Korea, Taiwan and Thailand) with hypertension, who are generally less responsive to isolated RASi [34]. The Prospective comparison of Angiotensin Receptor neprilysin inhibitor with Angiotensin receptor blocker MEasuring arterial sTiffness in the eldERly (PARAMETER) study is assessing the efficacy of LCZ696 versus olmesartan on central aortic haemodynamics and aortic stiffness in 432 patients (aged >60 years) [35]. The results are expected in 2015.

## NEPi IN HEART FAILURE

In early HF, NP levels increase to counteract salt and water retention. Over time the effects of NPs are negated by up-regulation of neurohormonal pathways, including RAS and the sympathetic nervous system, which cause further salt and water retention. Increasing levels of NPs with NEPi may help counteract up-regulation of these pathogenic pathways, when combined with RAS blockade.

The Omapatrilat Versus Enalapril Randomized Trial of Utility in Reducing Events (OVERTURE) trial randomized 5770 patients with New York Heart Association (NYHA) Classes II–IV HF to either omapatrilat or enalapril [36]. Non-significantly fewer patients treated with omapatrilat died or were hospitalized for HF compared with enalapril [914/2886 (32%) versus 973/2884 (34%); HR 0.94; 95% CI 0.86–1.03; P = 0.187] [36]. Angioedema was again more frequent with omapatrilat (0.8%) than enalapril (0.5%) but was less severe than in other trials [36].

The Prospective comparison of ARNi with ARB on Management Of heart failUre with preserved ejectioN fracTion (PARAMOUNT) trial randomized 301 patients with HF with preserved ejection fraction (HFpEF) to maximum tolerated daily doses of LCZ696 or valsartan [37]. The primary end point was change in NT-proBNP (as a marker of LV wall stress) from baseline to 12 weeks. NT-proBNP is a useful marker to study as it is not degraded by NEP, so any changes in NT-proBNP levels can still be used to assess disease severity in HF with NEPi [38]. In PARAMOUNT, greater reductions in NT-proBNP were seen with LCZ696 (ratio of change from baseline to 12 weeks 0.77; 95% CI 0.64–0.92; P = 0.005), in addition to improved NYHA class, BP and left atrial size. The drug was well tolerated, and although one case of angioedema occurred, it did not require hospitalization [37].

The Prospective comparison of ARNi with ACEi to Determine Impact on Global Mortality and morbidity in Heart Failure trial (PARADIGM-HF), the largest ever trial in HF with reduced ejection fraction (NYHA Classes II–IV) randomized 8436 patients to maximum daily tolerated doses of LCZ696 or enalapril [31, 39]. The primary outcome was a composite of time to first occurrence of either cardiovascular death or hospitalization for HF. Mean BP (mmHg) at baseline was 121/74 and LV ejection fraction 29% [39]. Mean serum creatinine at baseline was 99 μmol/L [mean estimated GFR (eGFR) 68 mL/min/1.73 m^2^] and 37% of participants had an eGFR <60 mL/min/1.73 m^2^ at enrolment [39].

The trial was closed early on the recommendation of the Data Monitoring Committee, having met the primary end point with overwhelming efficacy in favour of LCZ696 [40]. The full results of the trial are expected in the summer of 2014.

The Prospective comparison of ARni with Arb Global Outcomes in heart failure with preserved ejection fraction (PARAGON-HF) will soon start recruiting about 4300 patients with HFpEF and compare LCZ696 with valsartan. The primary outcome will be a composite of cardiovascular death and total (first and recurrent) hospitalizations for HF [38].

## NEPi IN CKD

The evidence for a potential role of NEPi in CKD comes from the study of NEPi in animal models of renal disease and the results of renal outcomes from trials in HF. However, no large-scale human trials have been conducted with this class of agents in a CKD cohort.

In an animal model of hypertension, long-term administration of omapatrilat led to dose-dependent reductions in BP and proteinuria that halted progression of glomerulosclerosis, tubulointerstitial fibrosis and renal injury [24]. In a 5/6 nephrectomy model, the anti-hypertensive and renoprotective effects of the VPI AVE7688 were compared with enalapril. Treatment was started once proteinuria and hypertension developed. AVE7688 greatly reduced proteinuria, glomerulosclerosis and tubulointerstitial fibrosis on renal biopsy [23]. Similar findings have also been observed in models of diabetic nephropathy [41]. AVE7688 increased renal synthesis of nitric oxide and decreased synthesis of endothelin-1 with reduced renal vasoconstriction and increased tubular ANP release [23]. In another 5/6 nephrectomy model, omapatrilat was administered at various time points following surgery [25]. Micropuncture studies demonstrated that omapatrilat led to greater reductions in SBP and capillary glomerular pressure [25]. The study also demonstrated reduced proteinuria and greater protection from renal injury with reduced glomerulosclerosis and delayed progression of renal disease with omapatrilat compared with ACEi-alone, which is likely to result from the effect on glomerular capillary pressure [25].

Candoxatrilat, an isolated NEPi, was compared with placebo in 24 patients with normal, moderately or severely reduced GFR in a cross-over study [42]. Compared with the placebo infusion, plasma ANP and urinary cGMP rose significantly after a 100-mg intravenous bolus of candoxatrilat. A marked natriuresis and diuresis occurred in all groups without changes in GFR or systemic BP [42].

In the Inhibition of Metallo Protease by Omapatrilat in a Randomized Exercise and Symptoms Study of Heart Failure (IMPRESS) trial (comparing omapatrilat with lisinopril), creatinine levels were reported as being raised more frequently in patients treated with lisinopril than omapatrilat (6.1 versus 1.8%, respectively; P = 0.009) [43]. Similarly, in the OVERTURE trial, worsening renal impairment occurred less frequently with omapatrilat (6.8 versus 10.1% with enalapril), despite including patients with moderate renal impairment (eligibility required serum creatinine <221 µmol/L at baseline) [36]. Over 36 weeks of follow-up of the PARAMOUNT trial (comparing LCZ696 with valsartan), eGFR declined to a lesser degree in the LCZ696 group (LCZ696, –1.6 mL/min/1.73 m² versus valsartan, –5.2 mL/min/1.73 m²; P = 0.007) [37]. However, albuminuria increased by 1 mg/mmol with LCZ696 compared with no change with valsartan (P = 0.02), but was very low at baseline [mean urine albumin:creatinine ratio (ACR) 2.0 mg/mmol] [37]. The PARADIGM-HF protocol includes renal-specific secondary end points: time to the composite of (i) 50% decline in eGFR relative to baseline, (ii) >30 mL/min/1.73 m² decline in eGFR relative to baseline eGFR of <60 mL/min/1.73 m² or (iii) progression to ESRD [31].

These studies highlight the potential advantages of combined NEPi/RASi in slowing the progression of CKD. However, the current data are indirect as they are based on animal models or HF populations. The UK Heart And Renal Protection III (UK HARP-III) trial (ISRCTN11958993) will compare LCZ696 against irbesartan in 360 patients with proteinuric CKD (urine ACR >20 mg/mmol and eGFR ≥20 <60 mL/min/1.73 m^2^). The trial will be the first test of an ARNi in a proteinuric population, and will assess the short-term safety and efficacy of LCZ696 in CKD with a primary outcome of the difference in change in measured GFR from baseline to 6 months between the two arms.

## CONCLUSION

NPs act as a potentially beneficial counter-regulatory system in states of excess RAS activation such as seen in hypertension, HF and CKD. In hypertension and HF, inhibition of neprilysin with LCZ696 has been shown to provide substantial clinical benefit. For patients with CKD, NEPi could be beneficial for two reasons: first, it may reduce the risk of CVD; second, it may retard the progression of CKD itself and delay the need for renal replacement therapy. A large randomized trial of an ARNi in a CKD population will be required to investigate this potential, but—if positive—such a trial would have a substantial impact on clinical practice.

## CONFLICT OF INTEREST STATEMENT

The UK HARP-III trial has been funded by Novartis. It will be conducted and interpreted independently of the principal funder. The Clinical Trials Service Unit and Epidemiological Studies Unit, which is part of the University of Oxford, has a staff policy of not accepting honoraria or consultancy fees.
